# Supplementary material for: Bias in nutrition-health associations is not eliminated by excluding extreme reporters in empirical or simulation studies
Source: eLife. 2023 Apr 5;12:e83616. doi: 10.7554/eLife.83616 (PMC10076015; doi:10.7554/eLife.83616)
Supplement: Figure 2—source data 1. [file elife-83616-fig2-data1.docx]

**Figure2-source data 1. Bias in associations between self-reported nutrition intakes and health outcomes**

| **Predictors** | **Outcomes** | Percent bias (%)^1,2^ | Percent remaining bias (%)^1,3^ |
| --- | --- | --- | --- |
| **Energy intake (Mcal/d)** | **Weight (kg)** | -84.80 (-101.70 to -67.90)* | -32.40 (-57.20 to -7.70)* |
|  | **Waist circumference (cm)** | -78.10 (-99.20 to -57.10)* | -33.10 (-63.70 to -2.50)* |
|  | **HR post fitness test (beat/min)** | 1.30 (-204.10 to 206.60) | 37.40 (-204.40 to 279.10) |
|  | **SBP (mmHg)** | -71.60 (-175.90 to 32.70) | -67.20 (-198.70 to 64.30) |
|  | **DBP (mmHg)** | -70.00 (-216.90 to 76.90) | 79.80 (-260.20 to 419.80) |
|  | **VO2 max (L/min)** | -96.30 (-305.70 to 113.00) | -131.00 (-583.80 to 321.90) |
| **Sodium intake**  **(g/d)** | **Weight (kg)** | 0.50 (-55.30 to 56.30) | 58.10 (-17.40 to 133.50) |
|  | **Waist circumference (cm)** | 6.60 (-57.30 to 70.40) | 31.40 (-50.90 to 113.80) |
|  | **HR post fitness test (beat/min)** | 130.20 (-157.10 to 417.50) | 230.20 (-180.70 to 641.20) |
|  | **SBP (mmHg)** | 38.00 (-1230.80 to 1306.80) | 14.20 (-1337.00 to 1365.50) |
|  | **DBP (mmHg)** | 94.80 (-546.50 to 736.00) | 220.70 (-658.20 to 1099.50) |
|  | **VO2 max (L/min)** | -99.50 (-360.90 to 161.80) | -241.80 (-808.90 to 325.20) |
| **Potassium intake (g/d)** | **Weight (kg)** | -41.00 (-167.20 to 85.30) | 90.50 (-119.60 to 300.60) |
|  | **Waist circumference (cm)** | -57.90 (-169.00 to 53.10) | -5.80 (-151.30 to 139.60) |
|  | **HR post fitness test (beat/min)** | 20.30 (-133.30 to 174.00) | 34.80 (-170.90 to 240.40) |
|  | **SBP (mmHg)** | -510.50 (-41767.00 to 40746.10) | -686.40 (-47014.60 to 45641.90) |
|  | **DBP (mmHg)** | -1951.10 (-253484.00 to 249581.80) | -3527.90 (-457168.90 to 450113.20) |
|  | **VO2 max (L/min)** | -28.70 (-126.30 to 68.90) | -14.90 (-150.20 to 120.30) |
| **Protein intake**  **(g/d)** | **Weight (kg)** | -33.60 (-74.00 to 6.90) | 2.80 (-53.10 to 58.60) |
|  | **Waist circumference (cm)** | -31.40 (-94.10 to 31.30) | -5.30 (-77.50 to 66.90) |
|  | **HR post fitness test (beat/min)** | -12.40 (-123.10 to 98.30) | -1.50 (-145.90 to 142.90) |
|  | **SBP (mmHg)** | -68.90 (-237.50 to 99.80) | -110.40 (-261.80 to 41.10) |
|  | **DBP (mmHg)** | -85.90 (-249.00 to 77.20) | -115.00 (-284.50 to 54.50) |
|  | **VO2 max (L/min)** | -95.40 (-237.80 to 46.90) | -125.90 (-321.80 to 70.00) |

Note: The numbers in parentheses are 95% CIs. The 95% CIs were computed using the jackknife method. ^*^: Significant association or bias. ^1^: Computed by jackknife estimation. ^2^: $\frac{\beta_{{NI}_{SR},HO}-\beta_{{NI}_{BIO},HO}}{\beta_{{NI}_{BIO},HO}}*100$ ^3^: $\frac{\beta_{{NI}_{G},HO}-\beta_{{NI}_{BIO},HO}}{\beta_{{NI}_{BIO},HO}}*100.$
