# Supplementary material for: Bias in nutrition-health associations is not eliminated by excluding extreme reporters in empirical or simulation studies
Source: eLife. 2023 Apr 5;12:e83616. doi: 10.7554/eLife.83616 (PMC10076015; doi:10.7554/eLife.83616)
Supplement: Figure 3—source data 1. [file elife-83616-fig3-data1.docx]

**Figure3-source data 1. Bias, MSE, and coverage probability in the simulation study**

| **NI** | **measurement** | **HO** | **Bias** | **MSE** | **CP** |
| --- | --- | --- | --- | --- | --- |
| EI | BIO | BW (kg) | 0.000009 | 0.000007 | 95.3 |
| EI | SR | BW (kg) | -0.01906 | 0.000368 | 0 |
| EI | G | BW (kg) | -0.01285 | 0.000173 | 11.1 |
| EI | BIO | WC (cm) | 0.000177 | 0.000006 | 95 |
| EI | SR | WC (cm) | -0.01324 | 0.000179 | 0 |
| EI | G | WC (cm) | -0.00887 | 0.000085 | 23.8 |
| EI | BIO | HR (beat/min) | -3.3E-05 | 0.000005 | 95.5 |
| EI | SR | HR (beat/min) | 0.00108 | 0.000003 | 88.2 |
| EI | G | HR (beat/min) | 0.000728 | 0.000006 | 94 |
| EI | BIO | SBP (mmHg) | 0.000125 | 0.000018 | 94.2 |
| EI | SR | SBP (mmHg) | -0.00359 | 0.000021 | 71.4 |
| EI | G | SBP (mmHg) | -0.00216 | 0.000023 | 92.4 |
| EI | BIO | DBP (mmHg) | 0.000039 | 0.000006 | 93.8 |
| EI | SR | DBP (mmHg) | -0.00286 | 0.000011 | 55.7 |
| EI | G | DBP (mmHg) | -0.00181 | 0.00001 | 90.6 |
| EI | BIO | VO2 max (L/min) | -0.00016 | 0.000111 | 94.1 |
| EI | SR | VO2 max (L/min) | -0.00204 | 0.000046 | 94.4 |
| EI | G | VO2 max (L/min) | -0.00148 | 0.000113 | 95.1 |
| SI | BIO | BW (kg) | 0.0341 | 0.647 | 94.8 |
| SI | SR | BW (kg) | -1.87091 | 5.1 | 67.4 |
| SI | G | BW (kg) | -1.89248 | 4.23 | 77.1 |
| SI | BIO | WC (cm) | -0.0086 | 0.458 | 95.5 |
| SI | SR | WC (cm) | -1.31196 | 2.84 | 74.2 |
| SI | G | WC (cm) | -1.3013 | 2.15 | 82.4 |
| SI | BIO | HR (beat/min) | 0.0149 | 0.345 | 94.3 |
| SI | SR | HR (beat/min) | 0.228872 | 0.727 | 94.3 |
| SI | G | HR (beat/min) | 0.201329 | 0.385 | 95.7 |
| SI | BIO | SBP (mmHg) | 0.0235 | 1.08 | 94.4 |
| SI | SR | SBP (mmHg) | -0.63884 | 2.7 | 92 |
| SI | G | SBP (mmHg) | -0.61277 | 1.45 | 92.2 |
| SI | BIO | DBP (mmHg) | 0.0247 | 0.318 | 95.2 |
| SI | SR | DBP (mmHg) | -0.47807 | 0.926 | 92.5 |
| SI | G | DBP (mmHg) | -0.49508 | 0.562 | 93 |
| SI | BIO | VO2 max (L/min) | 0.0943 | 6.25 | 94.8 |
| SI | SR | VO2 max (L/min) | -1.03963 | 13.7 | 93.7 |
| SI | G | VO2 max (L/min) | -1.11939 | 7.49 | 94.2 |
| PoI | BIO | BW (kg) | -0.0474 | 1.63 | 97.2 |
| PoI | SR | BW (kg) | -2.30023 | 8 | 70.6 |
| PoI | G | BW (kg) | -2.28744 | 6.86 | 78.3 |
| PoI | BIO | WC (cm) | 0.0455 | 1.22 | 95.3 |
| PoI | SR | WC (cm) | -1.53135 | 4.17 | 78.7 |
| PoI | G | WC (cm) | -1.51919 | 3.53 | 86.1 |
| PoI | BIO | HR (beat/min) | -0.00884 | 0.793 | 95.8 |
| PoI | SR | HR (beat/min) | 0.542101 | 1.52 | 91.2 |
| PoI | G | HR (beat/min) | 0.552034 | 1.1 | 92.1 |
| PoI | BIO | SBP (mmHg) | 0.0199 | 2.54 | 95.7 |
| PoI | SR | SBP (mmHg) | -0.1839 | 3.24 | 95.9 |
| PoI | G | SBP (mmHg) | -0.21543 | 2.59 | 95.6 |
| PoI | BIO | DBP (mmHg) | -0.0736 | 0.854 | 95.9 |
| PoI | SR | DBP (mmHg) | -0.20354 | 1.28 | 93.9 |
| PoI | G | DBP (mmHg) | -0.18457 | 0.882 | 93.7 |
| PoI | BIO | VO2 max (L/min) | 0.1842 | 18 | 92.9 |
| PoI | SR | VO2 max (L/min) | -3.77451 | 34.2 | 87.3 |
| PoI | G | VO2 max (L/min) | -3.86999 | 32.9 | 90.3 |
| PrI | BIO | BW (kg) | 0.000338 | 0.00179 | 94.7 |
| PrI | SR | BW (kg) | -0.11434 | 0.0155 | 30.8 |
| PrI | G | BW (kg) | -0.11572 | 0.0152 | 49.2 |
| PrI | BIO | WC (cm) | -0.00081 | 0.00133 | 95.1 |
| PrI | SR | WC (cm) | -0.0729 | 0.00706 | 52.1 |
| PrI | G | WC (cm) | -0.0732 | 0.00668 | 67.8 |
| PrI | BIO | HR (beat/min) | 0.000405 | 0.000893 | 95.2 |
| PrI | SR | HR (beat/min) | 0.0159 | 0.00114 | 93 |
| PrI | G | HR (beat/min) | 0.0159 | 0.00114 | 93.6 |
| PrI | BIO | SBP (mmHg) | 0.00346 | 0.00291 | 94.3 |
| PrI | SR | SBP (mmHg) | -0.0324 | 0.00401 | 91.7 |
| PrI | G | SBP (mmHg) | -0.0341 | 0.00406 | 92.6 |
| PrI | BIO | DBP (mmHg) | -0.00151 | 0.001 | 94.7 |
| PrI | SR | DBP (mmHg) | -0.0187 | 0.00137 | 90.3 |
| PrI | G | DBP (mmHg) | -0.0189 | 0.00136 | 92 |
| PrI | BIO | VO2 max (L/min) | -0.00197 | 0.0188 | 94.6 |
| PrI | SR | VO2 max (L/min) | -0.0561 | 0.0218 | 92.8 |
| PrI | G | VO2 max (L/min) | -0.0528 | 0.0216 | 93.8 |
