# Supplementary material for: Bias in nutrition-health associations is not eliminated by excluding extreme reporters in empirical or simulation studies
Source: eLife. 2023 Apr 5;12:e83616. doi: 10.7554/eLife.83616 (PMC10076015; doi:10.7554/eLife.83616)
Supplement: Supplementary file 1. [file elife-83616-supp1.docx]

**Supplementary File 1. List of abbreviation**

| **Abbreviation** | **Full form** |
| --- | --- |
| AARP | American Association of Retired Persons |
| ASA24 | Automated Self-Administered 24-Hour Dietary Assessment Tool |
| BIO | Biomarker |
| BW | Body weight |
| DBP | resting diastolic blood pressure |
| DHQ-II | Diet History Questionnaire |
| DLW | Doubly labelled water |
| EI | Energy intake |
| EE | Energy expenditure |
| FFM | Fat-free mass |
| FM | Fat mass |
| G | Goldberg cutoffs |
| HO | Health outcomes |
| HR | Heart rate |
| IDATA | Interactive Diet and Activity Tracking in American Association of Retired Persons |
| MSE | Mean squared error |
| NI | Nutrition intake |
| PoI | Potassium intake |
| PrI | Protein intake |
| SI | Sodium intake |
| SBP | Resting systolic blood pressure |
| SR | Self-reported |
| VO2 | VO_2_ max |
| WC | Waist circumference |
