# Supplementary material for: Bias in nutrition-health associations is not eliminated by excluding extreme reporters in empirical or simulation studies
Source: eLife. 2023 Apr 5;12:e83616. doi: 10.7554/eLife.83616 (PMC10076015; doi:10.7554/eLife.83616)
Supplement: Supplementary file 2. [file elife-83616-supp2.docx]

**Supplementary File 2. Estimated regression coefficients of the 3 analyses (per Mcal/day for EE and g/day for the others) with 95% CI computed by the jackknife method**

| **Predictors** | **Outcomes** | $\beta_{SR}$ | $\beta_{G}$ | $\beta_{BIO}$ | Percent bias (%)^1,2^ | Percent remaining bias (%)^1,3^ |
| --- | --- | --- | --- | --- | --- | --- |
| **Energy intake (Mcal/d)** | **Weight (kg)** | 3.09 (-0.38 to 6.56) | 13.72 (8.84 to 18.6)* | 20.31 (16.36 to 24.27)* | -84.80 (-101.70 to -67.90)* | -32.40 (-57.20 to -7.70)* |
|  | **Waist circumference (cm)** | 2.98 (0 to 5.96) | 9.12 (5.05 to 13.18)* | 13.63 (9.84 to 17.43)* | -78.10 (-99.20 to -57.10)* | -33.10 (-63.70 to -2.50)* |
|  | **HR post fitness test (beat/min)** | -2.64 (-4.98 to -0.31)* | -3.59 (-7.62 to 0.44) | -2.62 (-6.21 to 0.98) | 1.30 (-204.10 to 206.60) | 37.40 (-204.40 to 279.10) |
|  | **SBP (mmHg)** | 1.12 (-2.31 to 4.55) | 1.29 (-4.14 to 6.73) | 3.94 (-1.50 to 9.38) | -71.60 (-175.90 to 32.70) | -67.20 (-198.70 to 64.30) |
|  | **DBP (mmHg)** | 0.58 (-1.79 to 2.94) | 3.45 (-0.15 to 7.05) | 1.92 (-1.87 to 5.71) | -70.00 (-216.90 to 76.90) | 79.80 (-260.20 to 419.80) |
|  | **VO2 max (L/min)** | 0.18 (-9.10 to 9.45) | -1.44 (-20.01 to 17.12) | 4.76 (-7.70 to 17.21) | -96.30 (-305.70 to 113.00) | -131.00 (-583.80 to 321.90) |
| **Sodium intake**  **(g/d)** | **Weight (kg)** | 3.32 (1.67 to 4.97)* | 5.23 (3.16 to 7.29)* | 3.31 (2.14 to 4.47)* | 0.50 (-55.30 to 56.30) | 58.10 (-17.40 to 133.50) |
|  | **Waist circumference (cm)** | 2.50 (1.14 to 3.86)* | 3.08 (1.30 to 4.86)* | 2.34 (1.24 to 3.44)* | 6.60 (-57.30 to 70.40) | 31.40 (-50.90 to 113.80) |
|  | **HR post fitness test (beat/min)** | -1.53 (-2.84 to -0.22)* | -2.19 (-3.89 to -0.49)* | -0.66 (-1.48 to 0.15) | 130.20 (-157.10 to 417.50) | 230.20 (-180.70 to 641.20) |
|  | **SBP (mmHg)** | 1.17 (-0.80 to 3.15) | 0.97 (-1.81 to 3.74) | 0.86 (-0.94 to 2.66) | 38.00 (-1230.80 to 1306.80) | 14.20 (-1337.00 to 1365.50) |
|  | **DBP (mmHg)** | 0.56 (-0.61 to 1.73) | 0.93 (-0.62 to 2.48) | 0.29 (-0.54 to 1.13) | 94.80 (-546.50 to 736.00) | 220.70 (-658.20 to 1099.50) |
|  | **VO2 max (L/min)** | 0.01 (-4.98 to 5.01) | -2.99 (-10.40 to 4.42) | 2.12 (-2.00 to 6.24) | -99.50 (-360.90 to 161.80) | -241.80 (-808.90 to 325.20) |
| **Potassium intake (g/d)** | **Weight (kg)** | 1.16 (-1.42 to 3.74) | 3.75 (0.64 to 6.86)* | 1.97 (-0.22 to 4.16) | -41.00 (-167.20 to 85.30) | 90.50 (-119.60 to 300.60) |
|  | **Waist circumference (cm)** | 0.78 (-1.41 to 2.97) | 1.74 (-0.97 to 4.44) | 1.85 (-0.08 to 3.78) | -57.90 (-169.00 to 53.10) | -5.80 (-151.30 to 139.60) |
|  | **HR post fitness test (beat/min)** | -1.36 (-2.92 to 0.20) | -1.53 (-3.75 to 0.69) | -1.13 (-2.56 to 0.30) | 20.30 (-133.30 to 174.00) | 34.80 (-170.90 to 240.40) |
|  | **SBP (mmHg)** | -1.57 (-5.06 to 1.93) | -2.34 (-6.86 to 2.19) | 0.46 (-2.34 to 3.27) | -510.50 (-41767.00 to 40746.10) | -686.40 (-47014.60 to 45641.90) |
|  | **DBP (mmHg)** | -0.58 (-2.11 to 0.94) | -1.08 (-2.77 to 0.61) | 0.04 (-1.25 to 1.32) | -1951.10 (-253484.00 to 249581.80) | -3527.90 (-457168.90 to 450113.20) |
|  | **VO2 max (L/min)** | 4.75 (-2.03 to 11.53) | 5.67 (-3.51 to 14.85) | 6.67 (0.82 to 12.52)* | -28.70 (-126.30 to 68.90) | -14.90 (-150.20 to 120.30) |
| **Protein intake**  **(g/d)** | **Weight (kg)** | 0.10 (0.04 to 0.16)* | 0.15 (0.09 to 0.21)* | 0.15 (0.07 to 0.22)* | -33.60 (-74.00 to 6.90) | 2.80 (-53.10 to 58.60) |
|  | **Waist circumference (cm)** | 0.06 (0 to 0.13) | 0.09 (0.02 to 0.15)* | 0.09 (0.01 to 0.17)* | -31.40 (-94.10 to 31.30) | -5.30 (-77.50 to 66.90) |
|  | **HR post fitness test (beat/min)** | -0.03 (-0.07 to 0.01) | -0.03 (-0.08 to 0.01) | -0.03 (-0.07 to 0.01) | -12.40 (-123.10 to 98.30) | -1.50 (-145.90 to 142.90) |
|  | **SBP (mmHg)** | 0.02 (-0.06 to 0.09) | -0.01 (-0.09 to 0.07) | 0.06 (-0.02 to 0.14) | -68.90 (-237.50 to 99.80) | -110.40 (-261.80 to 41.10) |
|  | **DBP (mmHg)** | 0 (-0.03 to 0.04) | 0 (-0.04 to 0.040) | 0.03 (-0.02 to 0.07) | -85.90 (-249.00 to 77.20) | -115.00 (-284.50 to 54.50) |
|  | **VO2 max (L/min)** | 0 (-0.13 to 0.14) | -0.02 (-0.20 to 0.15) | 0.10 (-0.04 to 0.23) | -95.40 (-237.80 to 46.90) | -125.90 (-321.80 to 70.00) |

Note: The numbers in parentheses are 95% CIs. The 95% CIs were computed using the jackknife method. ^*^: Significant association or bias. ^1^: Computed by jackknife estimation. ^2^: $\frac{\beta_{{NI}_{SR},HO}-\beta_{{NI}_{BIO},HO}}{\beta_{{NI}_{BIO},HO}}*100$ ^3^: $\frac{\beta_{{NI}_{G},HO}-\beta_{{NI}_{BIO},HO}}{\beta_{{NI}_{BIO},HO}}*100.$
