# Supplementary material for: Bias in nutrition-health associations is not eliminated by excluding extreme reporters in empirical or simulation studies
Source: eLife. 2023 Apr 5;12:e83616. doi: 10.7554/eLife.83616 (PMC10076015; doi:10.7554/eLife.83616)
Supplement: Supplementary file 3. [file elife-83616-supp3.docx]

**Supplementary File 5. Statistical tests on the heteroskedasticity of the reporting error**

| **Nutrition Intake** | **P-value of the Goldfeld Quandt test** |
| --- | --- |
| Energy | 0.8666 |
| Sodium | 0.7374 |
| Potassium | 0.9686 |
| Protein | 0.8900 |
