# Supplementary material for: Bias in nutrition-health associations is not eliminated by excluding extreme reporters in empirical or simulation studies
Source: eLife. 2023 Apr 5;12:e83616. doi: 10.7554/eLife.83616 (PMC10076015; doi:10.7554/eLife.83616)
Supplement: Supplementary file 4. [file elife-83616-supp4.docx]

**Supplementary File 4. MSE of fitted polynomial functions for the reporting error**

|  | Order of the polynomial functions | | | | |
| --- | --- | --- | --- | --- | --- |
|  | **1** | **2** | **3** | **4** | **5** |
| Energy | 582729^*^ | 590639 | 598972 | 606127 | 614911 |
| Sodium | 2056408 | 1996615 | 1953706^*^ | 1960605 | 1974721 |
| Potassium | 1229786 | 1181882 | 1164808^*^ | 1170122 | 1184684 |
| Protein | 1371 | 1385 | 1336 | 1337 | 1322^*^ |

^*^ The order with the smallest MSE.
